# Facilitation of IL-22 production from innate lymphoid cells by prostaglandin E_2_ prevents experimental lung neutrophilic inflammation

**DOI:** 10.1136/thoraxjnl-2017-211097

**Published:** 2018-03-24

**Authors:** Jennifer M Felton, Rodger Duffin, Calum T Robb, Siobhan Crittenden, Stephen M Anderton, Sarah E M Howie, Moira K B Whyte, Adriano G Rossi, Chengcan Yao

**Affiliations:** Medical Research Council (MRC) Centre for Inflammation Research, Queen’s Medical Research Institute, The University of Edinburgh, Edinburgh, UK

**Keywords:** ARDS, cytokine biology, innate immunity, lymphocyte biology, airway epithelium

## Abstract

Acute lung injury is a neutrophil-dominant, life-threatening disease without effective therapies and better understanding of the pathophysiological mechanisms involved is an urgent need. Here we show that interleukin (IL)-22 is produced from innate lymphoid cells (ILC) and is responsible for suppression of experimental lung neutrophilic inflammation. Blocking prostaglandin E_2_ (PGE_2_) synthesis reduces lung ILCs and IL-22 production, resulting in exacerbation of lung neutrophilic inflammation. In contrast, activation of the PGE_2_ receptor EP4 prevents acute lung inflammation. We thus demonstrate a mechanism for production of innate IL-22 in the lung during acute injury, highlighting potential therapeutic strategies for control of lung neutrophilic inflammation by targeting the PGE_2_/ILC/IL-22 axis.

## Introduction

Acute lung injury (ALI)/acute respiratory distress syndrome (ARDS) is a life-threatening neutrophil-dominant disease with a significant morbidity and mortality for which effective therapies are currently lacking.^1^ Excessive pulmonary neutrophil recruitment mediates lung tissue damage, despite the beneficial role of neutrophils in promoting tissue repair.^2^ Therefore, there is a need for more effective medicinal agents for use in these severe and often lethal lung injury syndromes.^1^

Interleukin (IL)-22 is an IL-10 family member cytokine that is produced by both adaptive and innate immune cells. Recently, type 3 innate lymphoid cells (ILC3s) have been identified as the main source of innate IL-22.^3^ IL-22 is expressed in healthy human lung tissue, and patients with sarcoidosis and ARDS have decreased IL-22 levels.^3^ Innate IL-22 is protective against acute epithelial damage and inflammation in the lung as neutralisation of IL-22 exacerbates bacterial and viral infections and exogenous IL-22 attenuates bacterial pneumonias.^4^

Non-steroidal anti-inflammatory drugs (NSAID) inhibit cyclooxygenase activity and subsequent production of prostaglandins and are very widely used to manage many inflammatory conditions. Use of NSAIDs worsens lung bacterial infections and is a risk factor for severe sepsis.^5^ In contrast, prostaglandins including prostaglandin E_2_ (PGE_2_) are used for treating critical lung diseases with improved oxygenation and decreased pulmonary artery pressures.^6^ PGE_2_ exerts its biological actions through engagement of its four receptors, namely EP1–4. We have recently found that PGE_2_ promotes IL-22 production from both T cells and ILC3s.^7 8^ In this study, we have investigated the hypothesis that PGE_2_ inhibits acute lung neutrophilic inflammation through modulating lung ILC3 production of IL-22.

## Methods

Wild-type C57BL/6 mice were purchased from Harlan UK. *Rag1^-/-^* mice and mice with selective EP4 deficiency in T cells (Lck^Cre^EP4^fl/fl^ mice by crossing LckCre mice to EP4-flox mice)^9^ were maintained under specific pathogen-free conditions in accredited animal facilities. Mice were aged >7 weeks old at the beginning of use. All experiments were conducted in accordance with the UK Scientific Procedures Act of 1986 and had local institutional ethical approval. The ALI mouse model was induced by intratracheal injection of 10 µg of lipopolysaccharide (LPS) in combination with recombinant IL-22, EP2 and/or EP4 agonists, or indomethacin when indicated. After 24 hours, bronchoalveolar lavage (BAL) fluid and lung tissues were collected. Immune cells and cytokine production were measured by flow cytometry or ELISA. All data were expressed as mean±SD. The Student’s t-test or Mann-Whitney U test was used for statistical analyses by Prism V.6 (GraphPad) and p<0.05 was considered statistically significant.

## Results

**Figure 1:**
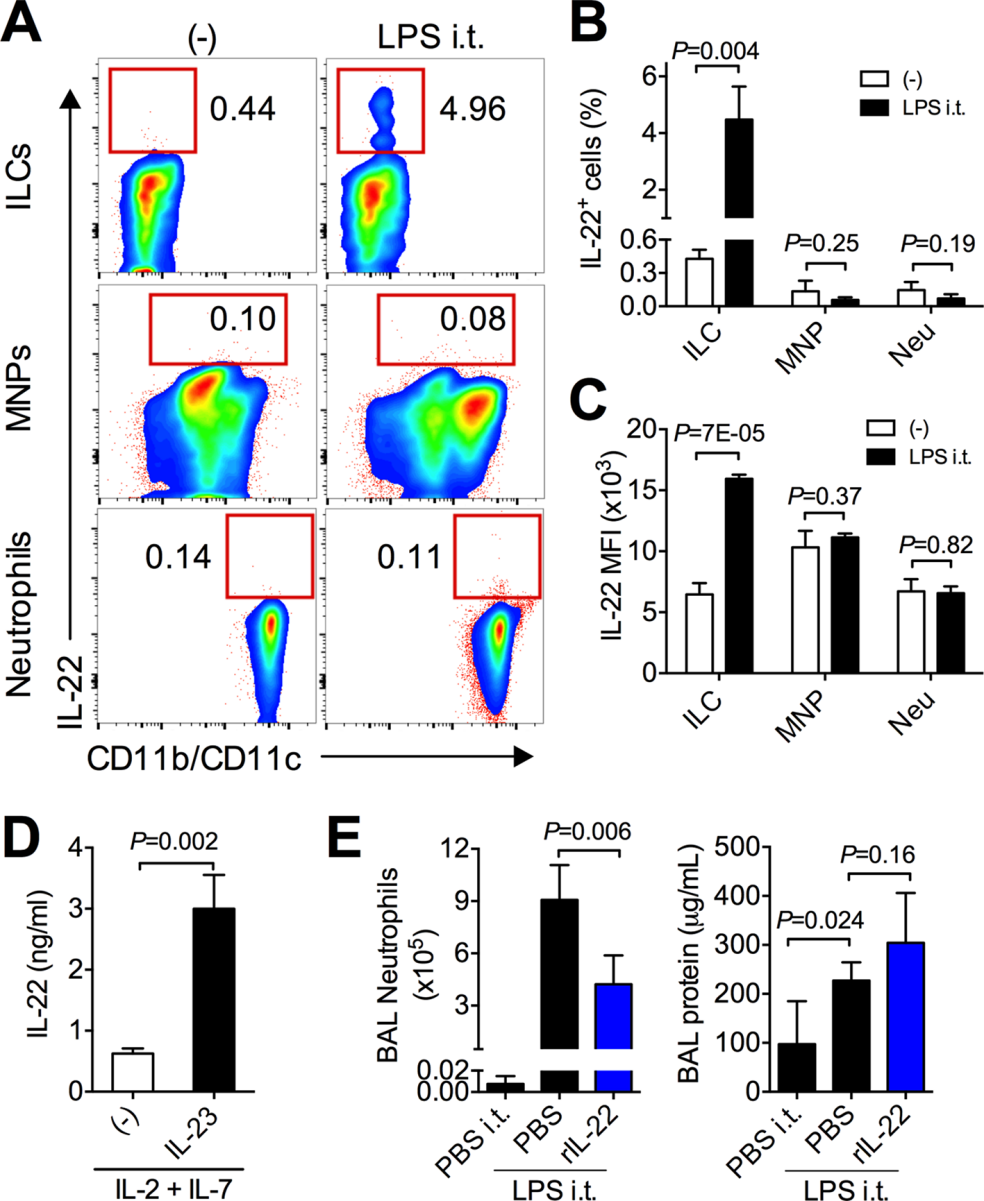
Production of IL-22 from ILCs in response to acute lung injury. (A) Representative flow cytometric dot plots for IL-22 expression in lung CD45^+^CD11b^−^CD11c^−^Ly-6G^−^ ILCs, CD45^+^CD11b/CD11c^+^Ly-6G^−^ MNPs and CD45^+^CD11b^+^Ly-6G^+^ Neu from *Rag1^-/-^* mice administered intratracheally with LPS for 24 hours (n=3 for each group). Lung cells were restimulated with IL-23 ex vivo for 4 hours before intracellular staining. (B, C) Percentages and MFI of IL-22^+^ cells. (D) Innate IL-22 production from lung immune cells isolated from naïve *Rag1^-/-^* mice and then cultured in vitro with indicated cytokines for 3 days (n=3). (E) Neutrophil numbers and total protein levels in BAL from mice administered intratracheally with PBS (n=3–4) or LPS in the absence (PBS, n=5) or presence of recombinant IL-22 (rIL-22, n=4). P values are calculated by Student’s t-tests. BAL, bronchoalveolar lavage; IL, interleukin; ILC, innate lymphoid cell; LPS, lipopolysaccharide; MFI, mean fluorescence intensity; MNP, mononuclear phagocyte; Neu, neutrophil; PBS, phosphate buffered saline.

To study the cellular sources of innate IL-22 in the lung, we administered a low dose (10 µg) of LPS to *Rag1^-/-^* mice, which have no adaptive T and B cells, and analysed IL-22 production from various lung immune cells 24 hours later using flow cytometry. In naïve mice, very few (~0.1%) CD11b^+^CD11c^+^Ly-6G^−^ mononuclear phagocytes (MNP) or CD11b^+^Ly-6G^+^ neutrophils produced IL-22, while ~0.4% CD11b^−^CD11c^−^Ly6G^-^CD90.2^+^ of ILCs expressed IL-22 (figure 1A,B and online supplementary figure). Intratracheal administration of LPS to the lung strikingly increased IL-22 production from ILCs by 10-fold to ~4% (n=3, p=0.004), but not from MNPs or neutrophils (figure 1A,B and online supplementary figure). The mean fluorescence intensity of IL-22 was significantly enhanced by LPS from ILCs (p=0.00007), suggesting that IL-22 production was also increased at the single cell level (figure 1C). Consistently, in response to appropriate stimulus lung innate immune cells produced IL-22 (figure 1D). IL-22 has been demonstrated to protect against inflammation at mucosal sites including the lung.^3^ To test whether IL-22 protects against ALI, we administered recombinant IL-22 intratracheally into mice immediately before LPS challenge. Exogenous IL-22 significantly suppressed LPS-induced acute lung neutrophilic inflammation (p=0.006) but had little effect on BAL total protein levels (figure 1E, n=3–5).

10.1136/thoraxjnl-2017-211097.supp1 Supplementary file 1

**Figure 2:**
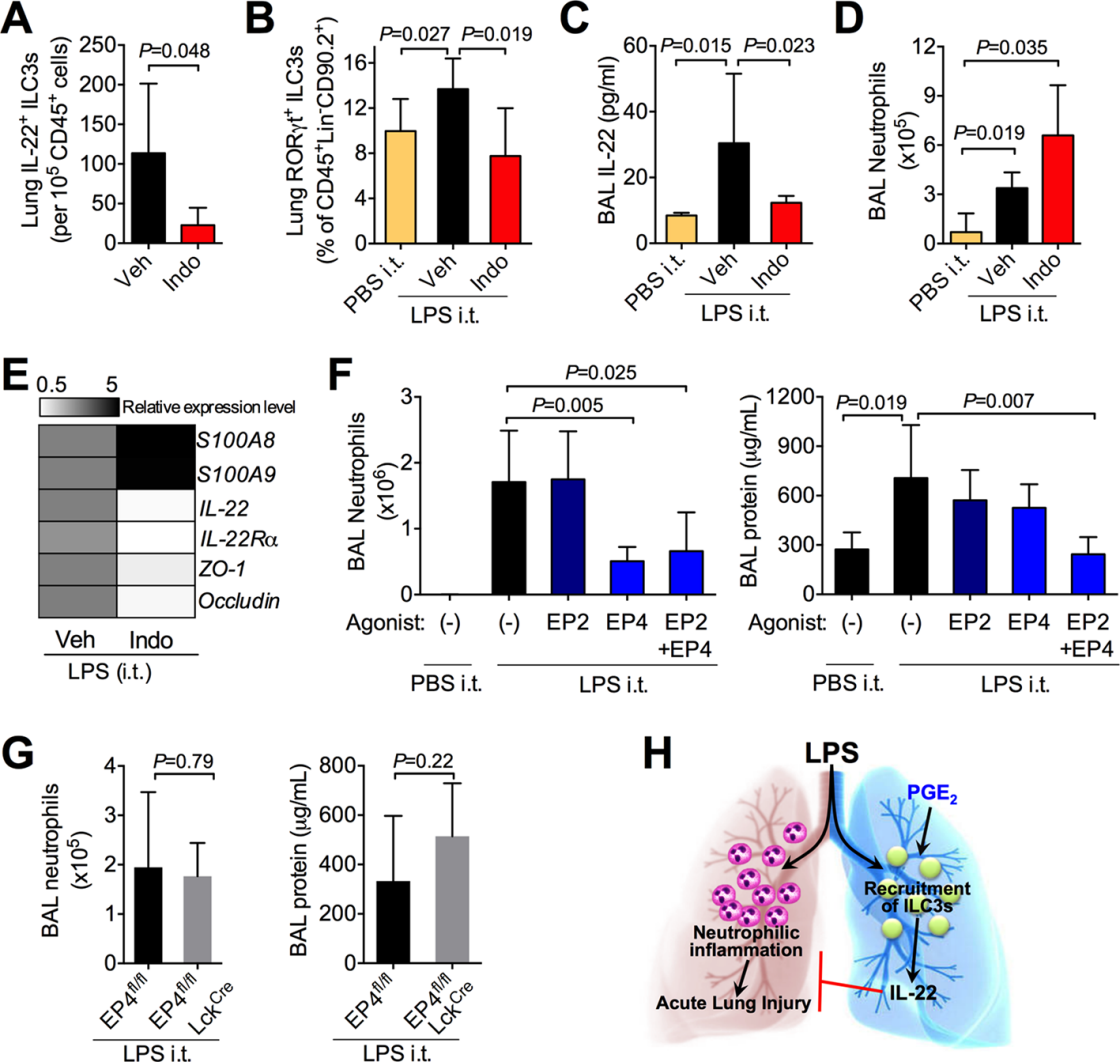
PGE_2_ promotes innate IL-22 production from lung ILC3s and inhibits acute lung injury. (A) IL-22-producing ILC3s in the lung from mice administered with vehicle or indomethacin for 5 days (n=4 for each group). Lung cells were restimulated with IL-23 ex vivo for 4 hours before staining. (B–E) Mice were administered with indomethacin (Indo) or vehicle control (Veh) via drinking water for 4 days and then challenged intratracheally with PBS or LPS. Lung tissue and BAL fluid were harvested at 24 hours after LPS or PBS challenge. Lung RORγt^+^ ILC3s (B, n=8, 8, 3), BAL IL-22 levels (C, n=8, 9, 3) and neutrophils (D, n=3, 4, 3) in the BAL were measured by flow cytometry. (E) Summarised gene expression in lung tissues determined by quantitative real-time PCR. Expression level for each gene in the vehicle group was set as 1 (n=4 for each group). (F) BAL neutrophil numbers and total protein levels in mice administered intratracheally with PBS or LPS plus an EP2 agonist (Butaprost), an EP4 agonist (L-902,688) or both (n=5–6 mice per group). (G) BAL neutrophil numbers and total protein levels in EP4^fl/fl^Lck^Cre^ (n=7) or control EP4^fl/fl^ (n=5) mice administered intratracheally with LPS. P values are calculated by Student’s t-tests or Mann-Whitney U tests. (H) Proposed mechanistic schematic diagram for PGE_2_ functions on restriction of acute lung neutrophilic inflammation through amplifying the ILC3/IL-22 pathway. BAL, bronchoalveolar lavage; IL, interleukin; ILC, innate lymphoid cell; LPS, lipopolysaccharide; PBS, phosphate buffered saline; PGE_2_, prostaglandin E_2_.

We have previously shown that PGE_2_ promotes intestinal ILC3 activation and IL-22 production.^7^ To examine whether PGE_2_ similarly regulates lung ILC3s and whether this leads to suppression of acute lung inflammation, we treated naïve mice with indomethacin, a cyclooxygenase inhibitor that inhibits endogenous PGE_2_ synthesis. Administration of indomethacin significantly reduced IL-22-producing ILC3s in the lung (figure 2A, n=4, p=0.048). In agreement with an increase in IL-22-producing ILC3s (figure 1A–C), LPS also induced accumulation of lung RAR-related orphan receptor gamma T (RORγt)^+^ ILC3s (p=0.027) and elevated BAL IL-22 levels (p=0.015, figure 2B,C). Coadministration of indomethacin decreased LPS-induced accumulation of RORγt^+^ ILC3s (p=0.019, n=4–8) and IL-22 production (p=0.023, n=3–9) in the lung (figure 2B,C). This reduction of ILC3s and IL-22 was associated with augmented LPS-induced acute lung neutrophilic inflammation (figure 2D, p=0.035, n=3–4). Inhibition of endogenous PGE_2_ downregulated genes responsible for IL-22 signalling (eg, IL-22, IL-22Rα) and lung epithelial barrier function (eg, ZO-1, Occludin) but upregulated S100A8/S100A9, which mediate neutrophilic lung inflammation (figure 2E). We then examined whether exogenous activation of PGE_2_ signalling prevented ALI using selective EP2 or EP4 agonists. The EP4, but not EP2, agonist markedly suppressed LPS-induced neutrophil accumulation (figure 2F, n=5–6, p=0.005), and coactivation of EP2 and EP4 receptors reduced total protein levels in the BAL (figure 2F, p=0.007), suggesting that PGE_2_-EP4 signalling limits neutrophilic lung inflammation. Because PGE_2_ also promotes adaptive IL-22 production from T cells,^8^ we examined whether T cells are involved in PGE_2_-modulated ALI using Lck^Cre^EP4^fl/fl^ mice. Deletion of EP4 in T cells did not affect LPS-induced neutrophil accumulation or total proteins in the BAL (figure 2G, n=5–7), further indicating that PGE_2_-EP4 signalling is important via a mechanism that stimulates innate IL-22 production from lung ILC3s.

## Discussion

Numerous experimental and clinical observations have shown that IL-22 plays a critical role in the control of mucosal injury and inflammation including pneumonia and ALI.^3^ This study further demonstrates that lung ILC3s are the major contributors of innate IL-22, which displays protective actions against neutrophilic inflammation in the airway. IL-22 has been reported to either protect against or promote allergic lung inflammation in the absence or presence of IL-17, respectively, suggesting that IL-22 functions in the lung in context-dependent manners.^10^ To augment IL-22 production or signalling, several approaches could be used, for example, application of IL-22 in protease-resistant forms via oral or intrapulmonary delivery or application of small chemical molecules that promote IL-22 production.^3^ Our findings show that PGE_2_ and its analogues (eg, EP4 agonists) could be used to catalyse the ILC3-IL-22 cascade and to facilitate the control of acute lung inflammation (figure 2H). Many lung cell types (eg, macrophages, damaged epithelial cells and neutrophils) can produce PGE_2_, which is essential to maintain ILC3 homeostasis and activation.^7^ Our findings highlight potential therapeutic strategies for control of lung neutrophilic inflammation by targeting the PGE_2_/ILC3/IL-22 pathway. It is envisioned that targeting this pathway by pharmacological intervention could have potential for the development of novel strategies for the treatment of lung inflammation especially in diseases where the neutrophil plays a prominent role.

Despite these important novel findings, we acknowledge this report has several limitations. First, the numbers of animals used in experiments were limited, so further validation of the findings may be needed by increasing mice numbers. Second, therapeutic effects of PGE_2_ analogues and rIL-22 on established neutrophilic lung inflammation induced by LPS or by other stimuli were not examined. Third, we have shown that exogenous IL-22 prevented ALI and that blocking PGE_2_ signalling reduced numbers of IL-22^+^ ILC3s in the lung. However, given the multiple roles of IL-22 in lung homeostasis as well as injury/inflammation, further studies to determine direct roles of endogenous IL-22 in ALI are warranted. Fourth, the protective actions of the PGE_2_/ILC3/IL-22 axis have not been examined in human patients with ALI/ARDS, although findings from our mouse studies could have important translational implications.
