# Supplementary material for: Facilitation of IL-22 production from innate lymphoid cells by prostaglandin E2 prevents experimental lung neutrophilic inflammation
Source: Thorax. 2018 Mar 24;73(11):1081–4. doi: 10.1136/thoraxjnl-2017-211097 (PMC6200127; doi:10.1136/thoraxjnl-2017-211097)
Supplement: Supplementary file 1 [file thoraxjnl-2017-211097supp001.pdf]

Research Letter

Facilitation of IL-22 production from innate lymphoid cells by prostaglandin E_2 prevents experimental acute lung injury

Jennifer M. Felton, Rodger Duffin, Calum T. Robb, Siobhan Crittenden, Stephen M. Anderton, Sarah E. Howie, Moira K. B. Whyte, Adriano G. Rossi*, Chengcan Yao*

Supplementary Methods

Reagents. Antibodies to mouse CD45 (clone 30-F11), CD11b (clone M1/70), CD11c (clone N418), CD90.2 (clone 30-H12), RORγt (clone B2D, eBioscience), Ly-6G (clone 1A8-Ly6g) and IL-22 (clone IL22J0P) were from eBioscience or Biolegend. Recombinant mouse IL-23 and IL-22 were purchased from Biolegend. PGE_2, Butaprost (EP2 agonist), and L-902,688 (EP4 agonist) were from Cayman, while LPS, indomethacin, phorbol myristate acetate (PMA), ionomycin were from Sigma.

Lung tissue digestion and flow cytometry. Lung tissue was cut into small pieces and digested with Liberase (0.2 mg/ml, Roche) and DNAse (25 ug/ml, Sigma) in Hanks buffer supplemented with 10% FBS at 37°C for 35 min to get single cell suspensions. For surface staining, cells were stained on ice with the Fixable Viability Dye eFluor® 780 (eBioscience) on ice for 30 min to exclude dead cells, washed and further incubated with indicated surface markers for another 30 min. For intracellular staining of IL-22 and RORγt, cells were

stimulated with IL-23 (20 ng/ml) for 4h in the presence of GolgiPlug (BD Bioscience). Cells were then fixed with Foxp3/Transcription Factor Fixation Buffer (eBioscience) followed by staining with anti-human/mouse IL-22 and Anti-Mouse ROR-γt in BD Perm/Wash Buffer (eBioscience). Flow cytometry was performed on an LSR Fortessa (BD Bioscience) and analyzed by FlowJo software (Tree Star).

Enzyme-linked immunosorbent assay (ELISA). IL-22 levels in culture supernatants and BAL fluids were detected using Ready-SET-Go!® ELISA kits for mouse IL-22 (eBioscience) according to the manufacturers' instructions.

Real-time PCR. RNA purification from homogenized lung tissues was performed by using the Rneasy Mini Kit (Qiagen). cDNA was obtained by reverse transcription using the High-capacity cDNA Reverse Transcription Kits (ABI). Samples were analyzed by real-time PCR GoTaq qPCR Master Mix (Promega) on the Applied Biosystem 7900HT Fast machine with respective primers published previously[7]. Expression was normalized to mouse glyceraldehyde-3-phosphate dehydrogenase (*Gapdh*) and presented as relative expression to vehicle control group by the $2^{-\Delta\Delta Ct}$ method.

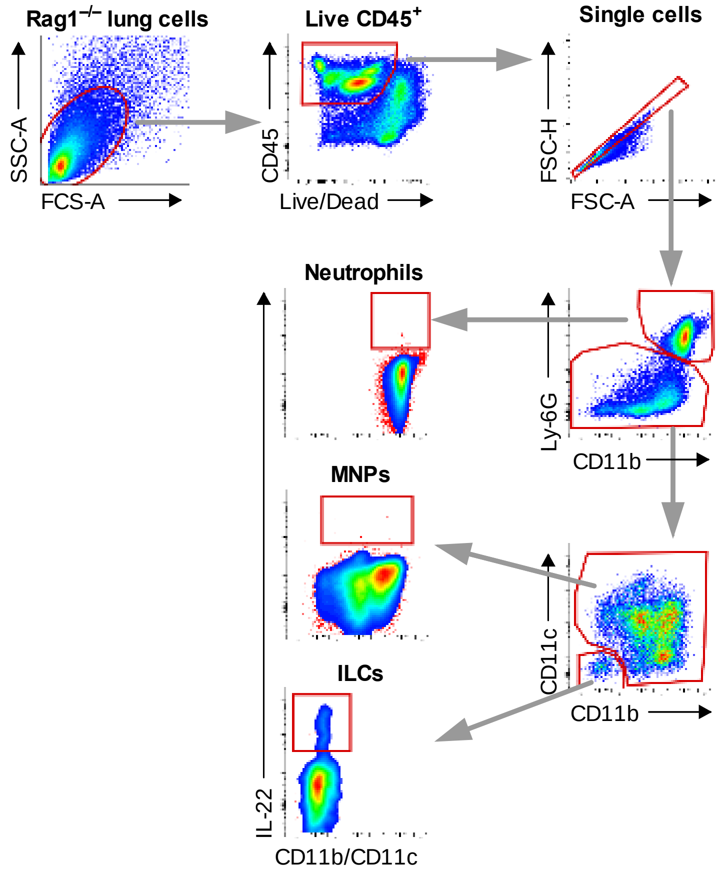

Supplementary Figure. Gating strategies for analysis of IL-22-producing cells in the lung. Lung cells were restimulated with IL-23 *ex vivo* for 4 h before staining.
